# The reaction of an iridium PNP complex with *para*hydrogen facilitates polarisation transfer without chemical change†
†Electronic supplementary information (ESI) available: Sample preparation, signal enhancements and raw data. CCDC 1026865. For ESI and crystallographic data in CIF or other electronic format see DOI: 10.1039/c4dt03088e



**DOI:** 10.1039/c4dt03088e

**Published:** 2014-11-20

**Authors:** Arthur J. Holmes, Peter J. Rayner, Michael J. Cowley, Gary G. R. Green, Adrian C. Whitwood, Simon B. Duckett

**Affiliations:** a Centre for Hyperpolarization in Magnetic Resonance , University of York , York Science Park , York , YO10 5NY , UK . Email: simon.duckett@york.ac.uk

## Abstract

The short lived pincer complex [(C_5_H_3_N(CH_2_P(^*t*^Bu)_2_)_2_)Ir(H)_2_(py)]BF_4_ is shown to be active for signal amplification by reversible exchange. This catalyst formulation enables the efficient transfer of polarization from *para*hydrogen to be placed into just a single molecule of the hyperpolarisation target, pyridine. When the catalysts ^1^H nuclei are replaced by ^2^H, increased levels of substrate hyperpolarization result and when the reverse situation is examined the catalyst itself is clearly visible through hyperpolarised signals. The ligand exchange pathways of [(C_5_H_3_N(CH_2_P(^*t*^Bu)_2_)_2_)Ir(H)_2_(py)]BF_4_ that are associated with this process are shown to involve the formation of 16-electron [(C_5_H_3_N(CH_2_P(^*t*^Bu)_2_)_2_)Ir(H)_2_]BF_4_ and the 18-electron H_2_ addition product [(C_5_H_3_N(CH_2_P(^*t*^Bu)_2_)_2_)Ir(H)_2_(H_2_)]BF_4_.

## Abstract 2

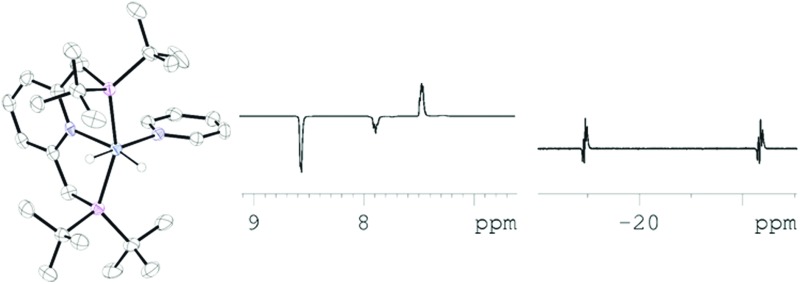
The pincer complex [(C_5_H_3_N(CH_2_P(^*t*^Bu)_2_)_2_)Ir(H)_2_(py)]BF_4_ is shown to be active for signal amplification by reversible exchange.

## Introduction

Nuclear magnetic resonance (NMR) spectroscopy finds widespread use in both chemistry and medicine despite the fact that it suffers from inherent low sensitivity due to the small energy differences that exist between the nuclear spin state orientations it probes.^1^ The net effect of this situation is that only 1 out of every 31 000 hydrogen atoms present in the sample contribute positively to the resulting signal in a room temperature ^1^H NMR spectrum that is recorded at 400 MHz (9.4 Tesla (T)). This challenge has resulted in the development of hyperpolarization methods to redistribute the nuclear spin state populations such that dramatically increased sensitivity is achieved.^2^ The potential to employ the resulting hyperpolarized signals in the field of clinical magnetic resonance imaging (MRI) has stimulated interest in this area.^3–5^ Rapid progress towards making the lifetime of the resulting magnetisation commensurate with clinical utility is helping to secure this methods future.^6–8^ Dynamic nuclear polarization (DNP) reflects one such hyperpolarisation approach that acts to increase the level of ^13^C polarisation in a range of materials.^9^ This increase has unlocked the use of pyruvate as a diagnostic MRI probe.^10^

Readily prepared *para*hydrogen (*p*-H_2_), one of the four nuclear spin isomers of dihydrogen, underpins another hyperpolarisation approach that has been widely utilised since Weitekamp's 1986 prediction.^11^ This method traditionally requires the reaction of a transition metal complex, or an unsaturated organic functionality, with *p*-H_2_. It actually involves the detection of these reaction products and relies on the fact that some of the newly introduced *p*-H_2_ derived spin order is preserved in the transformation.^12–14^ Such inorganic hydrogen addition products have yielded ^1^H NMR signals that reflect a 100% polarization level and are therefore 31 000 times larger than those normally visible when observed at 9.4 Tesla.^15^

**Scheme 1:**
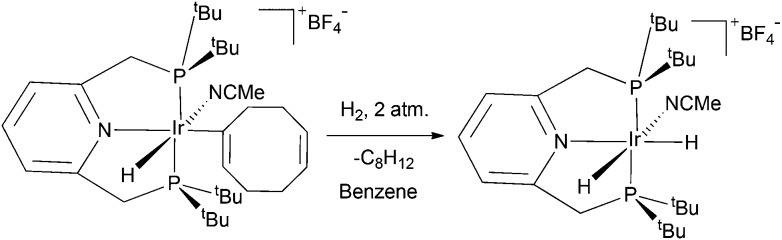
Reaction of [(C_5_H_3_N(CH_2_P(^*t*^Bu)_2_)_2_)IrH(C_8_H_11_)(NCCH_3_)]BF_4_ (**1**) with H_2_ forms the indicated *cis*-dihydride complex.^18^

In this study we utilize the reaction of 2,6-bis(di-*tert*-butylphosphinomethyl)-pyridine with [IrCl(COD)_2_]BF_4_ (COD = cyclooctadiene) to prepare the known PNP pincer^16,17^ complex [(C_5_H_3_N(CH_2_P(^*t*^Bu)_2_)_2_)IrH(C_8_H_11_)(NCCH_3_)]BF_4_ (**1**)^18^ (Scheme 1) and then explore its potential to act as a catalyst for signal amplification by reversible exchange (SABRE).^19^ This process involves the catalytic transfer of magnetism from *p*-H_2_ into what has been established to be a wide array of ligands which bind reversibly to transition metal centres.^20–23^ Subsequent equilibration of the metal-bound ligand with other molecules in bulk solution leads to the build up of these materials as hyperpolarised, and yet chemically unmodified, spin-probes that can be readily detected by NMR or MRI.^20,24,25^ The use of pincer complexes by Shaw^26^ has led to the development of many ligand systems,^27^ coupled with an array of novel reaction outcomes^28,29^ that include both the reduction of CO_2_ ^30^ and alkane dehydrogenation.^31,32^ Here we explore the fact that three metal coordination sites can be blocked by a pincer to improve the specificity of the SABRE hyperpolarisation transfer process. This has required the preparation of related ^2^H labelled derivatives.

## Results and discussion

### Reactions of [(C_5_H_3_N(CH_2_P(^*t*^Bu)_2_)_2_)Ir(H)_2_(NCCH_3_)]BF_4_ (**2**) with *para*hydrogen

In this study, **1** was first exposed to H_2_, to form the known dihydride complex [(C_5_H_3_N(CH_2_P(^*t*^Bu)_2_)_2_)Ir(H)_2_(NCCH_3_)]BF_4_ (**2**) as shown in Scheme 1.^18^ In *d*_4_-methanol, at 243 K, the ^1^H NMR spectrum of **2**, yields a dominant pair of hydride ligand signals at *δ* –19.28 and *δ* –20.87 as expected due to their chemical inequivalence. Consequently, these resonances would be expected to show *para*hydrogen induced polarisation (PHIP) activity.^33^ A smaller broad hydride resonance at *δ* –9.31 due to [(C_5_H_3_N(CH_2_P(^*t*^Bu)_2_)_2_)Ir(H_2_)(H)_2_]BF_4_ (**3**) was also seen, with the ratio of **2** : **3** in this ^1^H NMR spectrum being 6 : 1. Upon repeating this reaction with *p*-H_2_ at 273 K, the only PHIP enhanced signals appeared with anti-phase character in the alkyl region of the corresponding ^1^H NMR spectrum. The two hydride ligand signals of **2** and the broad resonance for **3** remained unpolarised. The observation of PHIP in the alkyl region of the NMR spectrum is consistent with the hydrogenation of COD to form cyclooctane, which is supported by its detection through GCMS analysis. The failure to see PHIP in the two hydride signals of **2** is surprising. Normally when *p*-H_2_ adds rapidly to a metal centre to produce such a dihydride complex, with inequivalent hydride ligands, strongly hyperpolarized hydride ligand signals result.^34–37^ We also see evidence for slow H/D exchange with the solvent such that free HD is detected as a 1 : 1 : 1 triplet at *δ* 4.54. Due to this exchange process, the hydride ligands appear with slightly reduced ^1^H signal intensities after 5 minutes at 273 K. Brookhart *et al*., have demonstrated a viable route to such H/D exchange exists through the acid catalysed transfer of a deuterium atom in a related system based on the pincer 2,6-bis(di-*tert*-butylphosphinito)pyridine, in this case the formation of a *trans*-dihydride iridium(iii) complex results.^38^

Interestingly when these measurements are repeated at 298 K, the hydride ligand signals of **2** and **3** are very broad in appearance. It is the presence of small amounts of **3** which causes the rapid quenching of the *p*-H_2_ reservoir that stops PHIP activity in this system. This species yields a single hydride resonance at *δ* –9.31, and a single CH_2_ spacer signal at *δ* 4.07 that is of equivalent area to the hydride signal. The ^*t*^Bu proton signal of **3** appears at *δ* 1.45. The *T*_1_ value for the *δ* –9.31 signal of **3** proved to be 24 ms at 233 K and is close to that reported for a related dihydrogen containing complexes.^38,39^ In contrast, the *T*_1_ values for the two hydride ligand signals of **2** are still large, at 260 and 274 ms respectively, and their values are indicative of an oxidative addition product.^40^ No exchange between **2** and **3** was indicated at 233 K.

**Scheme 2:**
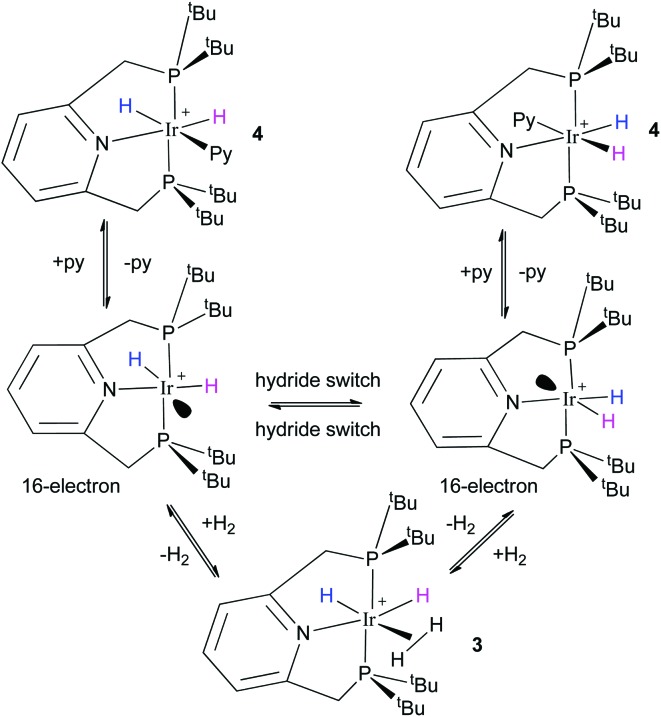
Ligand exchange processes exhibited by **4**. The complexes are cationic, and the hydride ligand colours illustrate the exchange processes assuming that the 16-intermediate has inequivalent hydrides and a vacant site (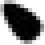).

When the same reaction is monitored in *d*_2_-dichloromethane, the same two hydride containing species are readily detected. The proportion of **3** increases when the H_2_ pressure is increased and the amount of CH_3_CN present in solution reduced. Unlike in *d*_4_-methanol, no H/D exchange occurs and consequently the hydride signals for **3** remain visible for extended periods of time. It proved possible under these conditions to detect magnetization transfer between H_2_, **2** and **3** *via* the exchange spectroscopy (EXSY)^41^ approach. Thus when the hydride signal of **3** is selected at 253 K, magnetization transfer into H_2_ and both of the hydride signals of **2** is visible. The magnetisation transfer proceeds at a rate of 8 s^–1^ into H_2_ and at a rate of 6.4 s^–1^ into **2** respectively. In contrast, magnetization transfer from H_2_ was observed to proceed first into **3** and then into **2** on a slower timescale. Furthermore, when **2** was probed in the same way, evidence for hydride site interchange within **2** and conversion into **3** was seen. Collectively, these data are consistent with the identity of **3** as the Ir(iii) complex [(C_5_H_3_N(CH_2_P(^*t*^Bu)_2_)_2_)Ir(H_2_)(H)_2_]BF_4_ which contains a set of rapidly interchanging hydride/dihydrogen ligands.^42^ It is this complex that provides the indirect route to H_2_ loss that is necessary for *p*-H_2_ introduction into **2**. We note that the related 16-electron dihydride complex [(C_5_H_3_N(CH_2_P(^*t*^Bu)_2_)_2_)Ir(H)_2_]PF_6_ has been observed previously in the absence of CH_3_CN and found as a minimum by DFT, where it contains inequivalent hydride ligands (see Scheme 2).^43^ In solution [(C_5_H_3_N(CH_2_P(^*t*^Bu)_2_)_2_)Ir(H)_2_]PF_6_ was found to yield a single hydride ligand signal at *δ* –28.65 and proved to be stable to H_2_ loss.^44^ The trapping of such a species with H_2_ would result in **3** as seen here; this reaction has been predicted by DFT to have a low barrier.^43^

### Reactions of [(C_5_H_3_N(CH_2_P(^*t*^Bu)_2_)_2_)Ir(H)_2_(py)]BF_4_ (**4**) with hydrogen

It is the rapid equilibration of **3** with H_2_ that leads to the destruction of *p*-H_2_. To overcome this challenge, a pale pink CD_3_OD solution of **1** was taken and exposed to both pyridine (22-fold excess based on **1**) and H_2_. The solution became colourless over minutes, and the resulting ^1^H NMR measurements at 298 K now revealed a pair of hydride ligand signals at *δ* –18.9 and –22.3 due to the pyridine adduct [(C_5_H_3_N(CH_2_P(^*t*^Bu)_2_)_2_)Ir(H)_2_(py)]BF_4_ (**4**). The identity of **4** was confirmed by NMR spectroscopy and mass spectrometry (see ESI†) and is directly analogous to **2** but it experiences an enhanced stability through the binding of pyridine rather than CH_3_CN.

When a similar CD_3_OD solution of **1** was taken and 0.625 equivalents of pyridine added under 3 atm. of H_2_, it proved possible to detect both **2** and **4** in a 1.2 : 1 ratio at 298 K. The line widths of the corresponding high field hydride signals at *δ* –18.9 and –19.3 for **4** and **2** respectively are now 40 and 112 Hz respectively. Line-broadening in NMR is a feature of fluxional behaviour.^45^ **2** exhibits the greatest increase in line-width and is therefore likely to be more reactive than **4**. The formation of **3** appears to be suppressed under these conditions but no PHIP is seen in the hydride ligands signals of either of these complexes when the corresponding reaction is repeated at 298 K under *p*-H_2_ and monitored by ^1^H NMR spectroscopy.

**Fig. 1:**
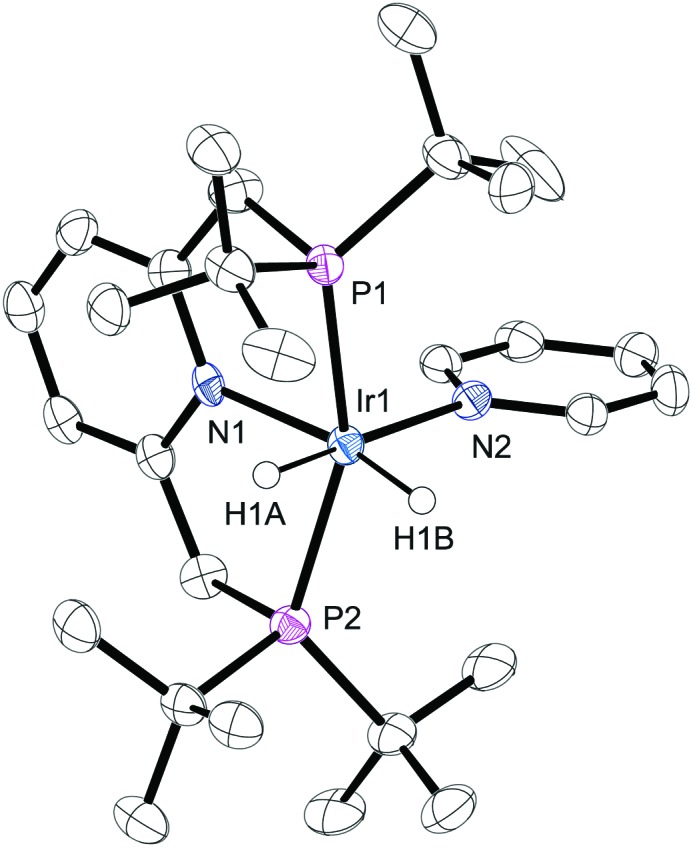
ORTEP plot of the cation **4**. Ellipsoids are set at the 50% probability level and the hydrogen atoms, solvent molecules and BF_4_^–^ counter ion are omitted for clarity.

The structure of **2** has been reported previously by Hermann^18^ and significant similarities exist between it and that of **4** determined here by X-ray crystallography (Fig. 1). For instance, the average Ir–P bond lengths in **2** and **4** are 2.314(4) Å and 2.312(6) Å respectively, and hence identical to within the measurements error. The corresponding P–Ir–P bond angles and Ir–N pincer bond lengths are also unchanged by replacement of CH_3_CN for pyridine. The Ir–NCCH_3_ bond length of **2** is shorter than that of the Ir–py bond length of **4** which is 2.189(4) Å by just 0.09(1) Å. When the Ir–py bond length is compared to those of [Ir(H)_2_(IMes)(py)_3_]Cl (2.180(3) Å) and [Ir(H)_2_(PCy_3_)(py)_3_]Cl (2.191(2) Å) no significant difference is seen.^20,24^ It might therefore be expected that the pyridine ligand in **4** is labile, as is the case for these IMes and PCy_3_ complexes.

A series of EXSY measurements were completed in order to probe both dihydrogen loss and pyridine ligand exchange by **4**; the relevant rate constant data are presented in Tables s1–s3.† Activation parameters from these measurements, determined over the temperature range 260 to 298 K by Eyring analysis revealed that for the pyridine loss pathway Δ*H*≠py-loss is just 79.0 ± 3 kJ mol^–1^. This is consistent with a relatively weak Ir–py bond strength when compared to that of [Ir(H)_2_(IMes)(py)_3_]Cl for which Δ*H*≠py-loss has been reported to be 93 ± 3 kJ mol^–1^.^20^ The Δ*S* ^≠^ value for this process of **4** is 50 ± 10 J K^–1^ mol^–1^ and matches that expected for dissociative ligand loss. Further evidence confirming the dissociative nature of this process is provided by the fact that saturation kinetic behaviour is seen at high pyridine excess (see ESI†).

In a separate experiment, the loss of H_2_ from **4** in CD_3_OH solution was monitored. In this study a 5.17 mM solution of **1** was employed where the pyridine concentration was 103.4 mM and the H_2_ pressure 3 atm. In this case Δ*H* ^‡^ for H_2_ loss proved to be 81 ± 4 kJ mol^–1^ and Δ*S* ^‡^ just 32 ± 13 J K^–1^ mol^–1^. The hydride ligands of **4** also proved to undergo competitive site interchange, where Δ*H* ^±^ is 88 ± 8 kJ mol^–1^ and Δ*S* ^≠^ 75 ± 30 J K^–1^ mol^–1^. Scheme 2 illustrates the reaction mechanism for these steps which start with the formation of the DFT predicted 16-electron complex, [(C_5_H_3_N(CH_2_P(^*t*^Bu)_2_)_2_)Ir(H)_2_]BF_4_, that contains a pair of inequivalent hydride ligands.^43^ [(C_5_H_3_N(CH_2_P(^*t*^Bu)_2_)_2_)Ir(H)_2_]BF_4_ can recombine with pyridine to reform **4** in a process where the relative hydride ligand orientations are retained, or it can reform **4** after the two hydride ligands have first switched their positions. It is the competitive formation of **3** and the resulting hydride/H_2_ ligand interchange, perhaps *via* sigma-CAM where the metal acts as the cam on which the ligands move, that facilities the observed exchange between free and bound H_2_ in **4** that is seen experimentally.^46^ The experimentally determined rate of hydride ligand site interchange in **4** was found to be *ca*. half that of pyridine loss in accordance with the process of hydride ligand switching being fast relative to the pyridine coordination. The observed rate of hydride ligand exchange into free H_2_ for **4** also increased with increase in H_2_ pressure; upon moving from 1 to 2 to 3 atm., the rate increased from 0.43 ± 0.01 to 0.69 ± 0.01 and then to 0.89 ± 0.01 s^–1^. Mechanistically, this rate increase corresponds to the more efficient trapping of [(C_5_H_3_N(CH_2_P(^*t*^Bu)_2_)_2_)Ir(H)_2_]BF_4_ with H_2_. Furthermore, the rate of hydride ligand exchange into free H_2_ from **4** proved to be reduced by added pyridine, even though the pyridine loss rate from **3** is itself unaffected because of its dissociative nature. These observations are fully consistent with the mechanism shown in Scheme 2.

These rate data serves to confirm that both SABRE and PHIP should be visible in the NMR spectra of **4** under a *p*-H_2_ atmosphere if the non-Boltzmann spin-population of *p*-H_2_ is not quenched too rapidly by **3**.

### Reactions of [(C_5_H_3_N(CH_2_P(^*t*^Bu)_2_)_2_)Ir(H)_2_(py)]BF_4_ (**4**) with *para*hydrogen

**Fig. 2:**
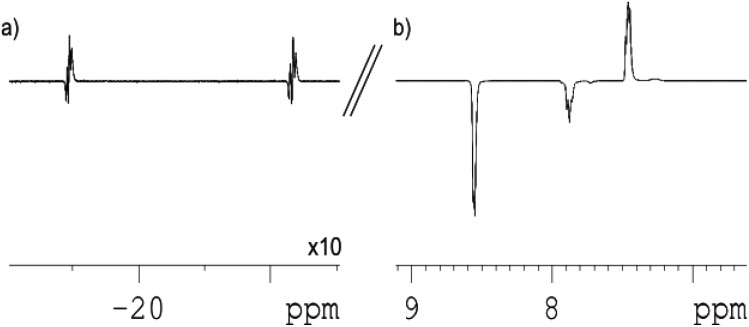
Hyperpolarized ^1^H NMR spectra of **4**, pyridine and *p*-H_2_ after polarisation transfer at 0.5 G: (a) hydride region; (b) aromatic region showing polarized signals of pyridine (×0.5 vertical expansion relative to (a)).

Under these conditions, when a sample of **1** containing pyridine is placed under *p*-H_2_ and monitored by high field ^1^H NMR spectroscopy at 298 K the hydride signals of **4** show PHIP (Fig. 2a). The strength of the detected hydride ligand signals, when compared to those of the thermally polarized system, allows an enhancement factor of 40 to be determined for them. This enhancement factor is substantial,^14^ and after a short period of time the hydride ligand signals diminish in intensity as the *p*-H_2_ in solution is consumed. The expected thermally polarized hydride ligand signals of **4** remain visible at this point in accordance with the stability of this complex.

When such a sample is initially examined, after being located outside the spectrometer, in a 0.5 G field, the SABRE effect is clearly visible (Fig. 2b) in the resulting ^1^H NMR spectra. Specifically, the resulting ^1^H NMR spectrum now contains an array of hyperpolarized NMR signals that in part correspond to the detection of free and bound pyridine. In this case, the free *ortho*-pyridyl proton resonance, which appears at *δ* 8.52, now shows a 6.5-fold signal enhancement relative to that of its normal, thermally equilibrated, level. This signal enhancement is a direct result of polarisation transfer from the hydride ligands of **4** into the *J*-coupling pyridine protons. The remaining *meta*- and *para*-proton signals of free pyridine are also enhanced, such that these three signals have relative intensities of 1 : –0.83 : –0.88 respectively.

**Fig. 3:**
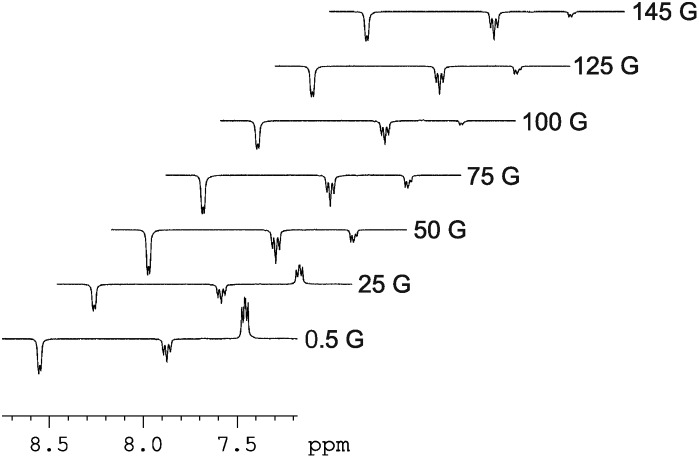
Series of hyperpolarized ^1^H NMR spectra showing how the appearance of the free pyridine signals change with change in the magnitude of the polarization transfer field experienced by **4**.

The signal intensity gain for the *ortho*-protons of free pyridine increases to 12.3-fold over their normal level when the magnetic field where polarization transfer takes place is increased to 65 G. This field change also results in all three of the free pyridine proton signals taking up the same phase with their relative intensities becoming 1 : 0.84 : 0.26 respectively. In these ^1^H NMR spectra, SABRE enhanced resonances are also seen at *δ* 9.30 (*ortho*), 8.06 (*para*) 7.24 (*meta*) which are due to the bound pyridine ligand of **4**, and at *δ* 7.64 and 7.91 for the pincer's pyridine protons. These data confirm that polarization flows from *p*-H_2_ first into the proton nuclei of the two ligands of **4** that lie *trans* to the two hydride ligands. Dissociation of bound pyridine ligand then accounts for the detection of hyperpolarised free pyridine in solution. It is noteworthy that the ^1^H NMR signal that is seen for the residual acetonitrile present in these samples does not receive any visible polarisation which is consistent with the failure to observed PHIP in **2**. Fig. 3 shows how the signal intensity changes for the free pyridine proton resonances as the polarization transfer field increases from 0.5 G to 145 G in steps of 25 G.

Warming these *d*_4_-methanol solutions of pyridine and **4** from 295 K to 310 K under *p*-H_2_ increases the level of polarization transfer into free pyridine. For example, the *ortho*, *para* and *meta* pyridine signals now show an 8, 20 and 35% increase in their relative signal strengths upon transfer at 310 K and 65 G when compared to those achieved at 295 K. This suggests that increasing the rate of ligand exchange by raising the temperature promotes polarisation transfer at 65 G. In contrast, the optimal temperature for transfer in a 0.5 G field is 300 K, with the corresponding signal intensities falling by 26, 18 and 27% respectively upon warming to 310 K. These changes suggest that lowering the rate of pyridine loss increases the transfer efficiency. Based on the reported theoretical studies dealing with SABRE, we know that polarisation transfer proceeds through the *J*-coupling network in a process that is moderated by the chemical shift difference between the interacting spins. The lifetime over which these interactions operate is also critical and controlled by the kinetic behaviour of the catalyst.^20^ At 298 K the rate of pyridine loss is 18 ± 4 s^–1^ and too slow for optimal transfer at 65 G but more closely matched for transfer at 0.5 G. At 310 K, the rate of pyridine loss is 66 ± 18 s^–1^ and more suited to transfer at 65 G; the maximum may not have been reached at this temperature. The optimum catalyst lifetime (the reciprocal of the ligand loss rate) must therefore vary with the value of the polarisation transfer field. Weakly interacting ligands should therefore be utilized in conjunction with catalysts such as **4** that contain inequivalent hydride ligands and be studied at high polarisation transfer fields. It is clear, however, that the underlying efficiency of **4** is less than that which has been reported for [Ir(H)_2_(IMes)(py)_3_]Cl where resonance enhancement values of >3000 have been seen.^20^

At 298 K, increasing the pressure of *p*-H_2_ from 1, to 2 and then 3 atm. successfully increases the level of free pyridine polarization by 1.7 and 2.0 times respectively. This process has no effect on the lifetime of **4** but does clearly increase the effective level of *p*-H_2_ derived polarisation that can be utilised during the catalytic step. It is also possible to maintain an increased level of *p*-H_2_ in solution during these processes by reducing the metal complex concentration. This effect works by reducing the concentration of **3** and hence its ability to reduce the purity of *p*-H_2_. This change therefore ensures that the maximum level of spin polarization associated with *p*-H_2_ is made available for sharing during the reaction.

### Promoting SABRE *via* deuterium labelling

Other studies have revealed that when the spin polarization associated with *p*-H_2_ is actually shared with fewer proton nuclei in the substrate larger signal enhancements result.^20,21^ In other words, a lack of transfer specificity during the SABRE process can actually be viewed as being detrimental to catalysis because it corresponds to what is effectively by-product formation. A simple synthetic route to overcome this challenge is to share the *p*-H_2_ spin order with fewer protons through deuterium labelling. This concept has been exemplified previously by using mixtures of *d*_5_-pyridine and *h*_5_-pyridine, or indeed partially labelled targets.^20,21^ When, *d*_5_-pyridine is used here, the *p*-H_2_ derived spin order that enters **4** transfers into its ^1^H-ligand receptor resonances which correspond to the pincer signals at *δ* 7.64 and 7.91 and these are strengthened. Furthermore, as the *d*_5_-pridine is not 100% labelled, the residual *ortho* proton signal at *δ* 8.4 also appears as an enhanced signal. In view of the fact that for SABRE to operate, polarisation transfer must take place in low field the enhanced signals seen for these groups vanish, as expected, in the next scan. This gain in ^1^H signal strength is the direct result of the less efficient transfer of polarization into the ^2^H nuclei which arises because they are very far apart in terms of frequency. We hypothesized that this effect could be used in reverse to favour polarization transfer into the weakly bound pyridine ligand if the pincer was deuterated. We therefore prepared the corresponding complexes ***d*_4_-1** and ***d*_7_-1** using the deuterated pincer ligands [(C_5_H_3_N(CD_2_P(^*t*^Bu)_2_)_2_)] and [(C_5_D_3_N(CD_2_P(^*t*^Bu)_2_)_2_)] respectively.

**Scheme 3:**
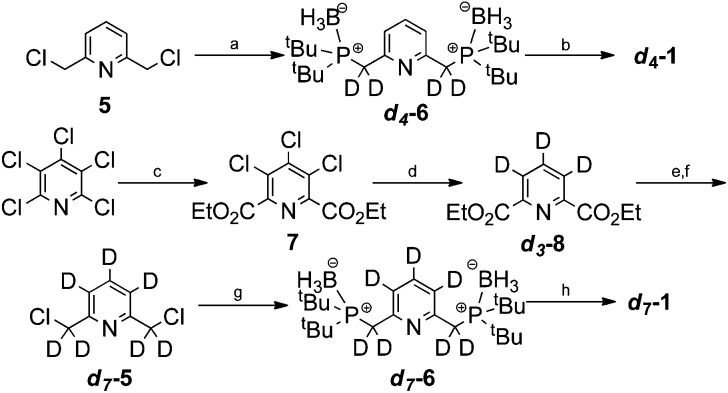
Synthesis of ***d*_4_-1** and ***d*_7_-1**. (a) (^*t*^Bu)_2_PHBH_3_, 40% NaOD(D_2_O), TBAB, toluene, rt, 16 h (98%, 95%D), (b) (i) HBF_4_·OEt_2_, MeOD, 80 °C, 16 h (ii) DIPA polymer bound, MeOD, rt, 1 h (iii) [Ir(COD)_2_]BF_4_ (47%), (c) CO(g) (5 Bar), NaCO_3_, Pd(OAc)_2_, DPPB, EtOH, 100 °C, 16 h (55%), (d) 5% Pd/C, D_2_(g), Et_3_N, D_2_O/THF, rt, 5 h (82%, 98% D), (e) NaBD_4_, MeOD, 65 °C, 2 h (86%, 98% D), (f) SOCl_2_, THF, 65 °C, 1 h (93%), (g) (^*t*^Bu)_2_PHBH_3_, 40% NaOD(D_2_O), TBAB, toluene, rt, 16 h (99%), (h) (i) HBF_4_·OEt_2_, MeOD, 80 °C, 16 h (ii) DIPA-polymer bound, MeOD, rt, 1 h (iii) [Ir(COD)_2_]BF_4_ (57%).

The synthesis of ***d*_4_-1** began by alkylation of borane di(*tert*-butyl)phosphine complex with 2,6-bis(chloromethyl)pyridine (**5**) as shown in Scheme 3.^47^ This gave the phosphine–borane complex ***d*_4_-6** in 98% yield with 95% deuterium incorporation. We then developed a telescoped procedure to ***d*_4_-1** in 47% yield over three steps; borane deprotection was effected using HBF_4_·OEt_2_ in MeOD, liberation of the free bisphosphine was achieved on polymer supported diisopropylamine (PS-DIPA) and finally complexation with [Ir(COD)_2_]BF_4_ under reported conditions.^48^ Importantly, this deprotection–complexation procedure proceeded with no observable deuterium–hydrogen exchange according to both mass spectrometry and NMR analysis.

Pentachloropyridine provided a suitable starting material for the synthesis of ***d*_7_-1**. First, carbonylation gave the trichlorodiester **7** in 55% yield.^49^ We found that efficient deuteration of the pyridine ring could then be achieved by D_2(g)_ and 5% Pd/C in D_2_O/THF. This furnished the *d*_3_-diester ***d*_3_-8** in 82% yield and with 98% deuterium incorporation. Subsequent reduction and chlorination gave ***d*_7_-5** in 80% yield over two steps. The synthesis was then completed under the analogous conditions to those used to prepare ***d*_4_-1**.

The level of SABRE seen in the NMR signals of pyridine was found to change as a consequence of this ^2^H labelling at both 0.5 G, and 65 G relative to that achieved with **1**. For ***d*_4_-1** the corresponding enhancement values for the *ortho* protons were 7 and 16 fold respectively, whilst for ***d*_7_-1** they were 9 and 16 respectively. These values reflect an increase in efficiency of 35% at 65 G.

## Conclusions

In this paper we have demonstrated that the pincer complex [[(C_5_H_3_N(CH_2_P(^*t*^Bu)_2_)_2_)Ir(H)_2_(py)]BF_4_ (**4**) is able to act as a SABRE catalyst. In the case of pyridine a 12.3-fold signal increase for the *ortho* proton resonance has been observed which is significant in terms of an NMR sensitivity gain. Both the ^1^H nuclei of the pincer pyridine arm and of the pyridine ligand receive a share of the polarisation during this process. ^2^H-labelling of the pincer pyridine arm has been used to suppress unnecessary magnetization wastage into it and thereby further increase the catalysts efficiency in polarising free pyridine by 35%. When *d*_5_-pyridine is used a similar improvement in the signal strengths of the pincer pyridine arm proton resonances results and now the catalyst itself can be readily viewed through the detection of SABRE enhanced signals. In the case of **4**, the ligand exchange rate constants are too slow for optimal SABRE transfer at 65 G and warming improves SABRE efficiency. Surprisingly, the reverse is true at 0.5 G where lower temperatures deliver improved catalysis. The lifetime of **4** is therefore important and it would appear that for weakly interacting ligands optimal polarisation transfer can be obtained through transfer in relatively high fields.

In **4**, only one molecule of the hyperpolarization target, in this case pyridine, is bound at any one time. This compares to three in [Ir(H)_2_(IMes)(py)_3_]Cl,^20^ and it might therefore be expected that such complexes could be made more effective for SABRE if their ligand exchange rates were further optimised. In this case, it is the presence of the dihydrogen complex **3** that leads to the efficient relaxation of *p*-H_2_ which helps preclude good activity. As the formation of such a species can be avoided by increasing the electron density on the metal centre, it might be expected that this challenge can be overcome.^50^ In view of the fact that this simple hyperpolarization method rapidly produces highly visible contrast agents which could be used in MRI diagnosis there is a clear need for the development of such magnetization transfer catalysts.^9^

## Experimental

### Materials and methods

All experimental procedures were performed under an atmosphere of nitrogen gas using standard Schlenk line techniques or in an M-Braun glove box. All solvents were dried using an M-Braun solvent system or distilled from the appropriated drying agent under N_2_. Deuterated *d*_4_-methanol, and *h*_5_-pyridine were obtained from Sigma-Aldrich, *d*_5_-pyridine was obtained from Cambridge Isotopes and used as supplied.

### Synthetic procedures

[(C_5_H_3_N(CH_2_P(^*t*^Bu)_2_)_2_)IrH(C_8_H_11_)(NCCH_3_)]BF_4_ (**1**) was prepared as according to published methods.^18^ 2,6-Dimethyl-3,4,5-trichloropyridine dicarboxylate (**7**), *d*_3_-2,6-diethyl pyridine dicarboxylate (***d*_3_-8**), *d*_7_-2,6 pyridine dimethanol, *d*_7_-2,6-bis(chloromethyl) pyridine (***d*_7_-5**), *d*_7_-2,6-bis(di-*tert*-butylphosphinomethyl)pyridine di-borane complex (***d*_7_-6**), [(C_5_D_3_N(CD_2_P(^*t*^Bu)_2_)_2_)IrH(C_8_H_11_)(NCCH_3_)]BF_4_ ***d*_7_-1**, *d*_4_-2,6-bis(di-*tert*-butylphosphinomethyl)pyridine di-borane complex (***d*_4_-6**), [(C_5_H_3_N(CD_2_P(^*t*^Bu)_2_)_2_)IrH(C_8_H_11_)(NCCH_3_)]BF_4_ ***d*_4_-1** and *h*_7_-2,6-bis(di-*tert*-butylphosphinomethyl)pyridine di-borane were prepared as described in the ESI.†

### Crystallography

Crystals of **4** were grown from a concentrated solution in methanol. The sample was layered with benzene and the solvent allowed to evaporate slowly. A suitable crystal was selected and mounted on a SuperNova, Single source Eos diffractometer. The crystal was kept at 109.9 K. The structure was solved using Olex2,^51^ and the olex2.solve structure solution program and refined within the XL refinement package using least squares minimization.^52^
