# Supplementary material for: The reaction of an iridium PNP complex with parahydrogen facilitates polarisation transfer without chemical change
Source: Dalton Trans. 2014 Nov 20;44(3):1077–83. doi: 10.1039/c4dt03088e (PMC4295814; doi:10.1039/c4dt03088e)
Supplement: Supplementary file 1 [file DT-044-C4DT03088E-s001.pdf]

Electronic Supplementary Material (ESI) for Dalton Transactions.
This journal is © The Royal Society of Chemistry 2014

Supplementary information.

The reaction of an Iridium PNP complex with *para*hydrogen facilitates polarization transfer without chemical change.

Arthur J. Holmes, Peter J. Rayner, Michael J. Cowley, , Gary G. R. Green, Adrian C. Whitwood and Simon B. Duckett*

Contents

1. General conditions: 3
2. Synthetic procedures 3
3. Sample preparation: 7
4. Kinetic measurements of the exchange of H_2 and pyridine in **4** 7
5. Effect of hydrogen pressure. 8
6. Effect of pyridine concentration on the pyridine on hydride ligand exchange rates. 10
7. Quantification of polarisation: 12
8. Effect of transfer field and temperature 12
9. Characterisation data for **4** 13
10. X-Ray crystal structure for **4** 13

1. General conditions

All experimental procedures were performed under an atmosphere of nitrogen gas using standard Schlenk line techniques or in an M-Braun glove box. All solvents were dried using an M-Braun solvent system or distilled from the appropriated drying agent under N_2. Deuterated d_4-methanol, and h_5-pyridine were obtained from Sigma–Aldrich, d_5-pyrdine was obtained from Cambridge isotopes and used as supplied.

2. Synthetic procedures

$[(C_5H_3N(CH_2P(tBu)_2)_2)IrH(C_8H_{11})(NCCH_3)]BF_4$ **(1)** was prepared as accoring to publised methods[1].

2,6-Dimethyl-3,4,5-trichloropyridine dicarboxylate[2]

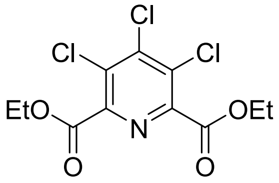

Pentachloropyridine (4.58 g, 18.0mmol, 1.0 eq.), $Pd(OAc)_2$ (262mg, 1.17mmol, 6.5 mol%), 1,4-bis(diphenylphosphino)butane (515mg, 1.21mol, 6.7 mol%), and Na_2CO_3 (2.00 g, 18.9mmol, 1.05 eq.) in EtOH (50 mL) were sealed in a 300 mL pressure reactor and purged with N_2.Then, the reactor was pressurised to 5bar with CO and the reactor was heated at 100 °C for 20 h. During this time a small pressure drop wasobserved. The reactor was cooled, vented and the contents filtered through Celite®. The filtrate was concentrated under reduced pressure to give the crude product. Purification by flash column chromatography on silica with 95:5-80:20 hexane-EtOAc as eluent gave 2,6-Dimethyl-3,4,5-trichloropyridine dicarboxylate(3.24g, 55%) as a colourless oil, R_f (9:1 hexane-EtOAc) 0.2; ^{1}H NMR (500 MHz, $CDCl_3$)δ4.45 (q, J = 7.0 Hz, 2H, CH_2), 1.40 (t, J = 7.0 Hz, 3H, Me); ^{13}C NMR (100.6 MHz, $CDCl_3$) δ163.1 (C=O), 147.4, 144.2, 131.2, 62.9 (CH_2), 14.0 (Me); MS (ESI) m/z 326 [$(M + H)^+$, 100], 328 [$(M + H)^+$, 100], 330 [$(M + H)^+$, 25]; HRMS m/z calcd for $C_{11}H_{10}{}^{35}Cl_3NO_4$ $(M + H)^+$325.9754, found 325.9759 (+0.5 ppm error). Spectroscopic data consistent with those reported in the literature.[2]

***d*$_3$-2,6-Diethyl pyridine dicarboxylate**

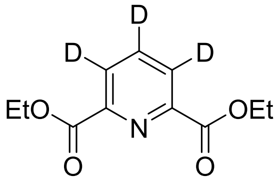

5% Pd/C (75 mg, 10 wt%) in D_2O (8 mL) was stirred under a D_2(g) atmosphere for 1 h. Then, a solution of 2,6-Dimethyl-3,4,5-trichloropyridine dicarboxylate(750 mg, 2.31 mmol, 1.0 eq.) and Et_3N (1.0 mL) in THF (3 mL) was added dropwise. The resulting suspension was stirred at rt for 5 h. Then, the suspension was filtered through Celite® and the filtrate was extracted with EtOAc (3 x 15 mL). The combined organic layers were dried (Na_2SO_4) and concentrated under reduced pressure to give the crude product. Purification by flash column chromatography on silica using 8:2 - 4:6 hexane-EtOAc as eluent gave d_3-diethyl pyridine dicarboxylate (428 mg,82%, 98%D incorporation) as a colourless needles,mp 41-43 °C (lit.,[3]45-46 °C);R_f(1:1 hexane-EtOAc) 0.2; ^{1}H NMR (500 MHz, $CDCl_3$)δ8.23 (s, 0.03H), 7.97 (s, 0.02H), 4.45-4.40 (m, 2H), 1.41-1.37 (m, 3H); ^{13}C NMR

(125.8 MHz, $CDCl_3$) δ 164.6 (C=O), 148.5, 137.7 (t, J = 25 Hz), 127.6 (t, J = 25 Hz), 62.2 (CH_2), 14.2 (Me);MS (ESI) m/z 227 [$(M + H)^+$, 100]; HRMS m/z calcd for $C_{11}H_{10}D_3NO_4$ $(M + H)^+$227.1111, found 227.1113 (+0.2 ppm error). Spectroscopic data consistent with those reported in the literature.[3]

d_7-2,6 pyridine dimethanol

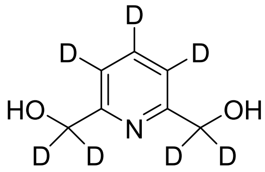

$NaBD_4$ (238 mg, 5.68mmol, 4.0 eq.) was added portion wise to a stirred solution of d_3-diethyl pyridine dicarboxylate(320 mg, 1.42 mmol, 1.0 eq.) in MeOD (7 mL) under N_2. The resulting solution was stirred at 65 °C for 2 h. Then, the solution was cooled to rt and concentrated under reduced pressure to give the crude product. Purification by flash column chromatography on silica using 95:5 - 7:3CH_2Cl_2-MeOH as eluent gave d_7-pyridine dimethanol (178 mg, 86% yield, 98%D incorporation) as a white solid, mp110-112°C (lit.,[4] 114-115 °C);R_f (8:2 CH_2Cl_2-MeOH) 0.15; ^{13}C NMR (100.6 MHz, $CDCl_3$) δ158.2, 137.6(t, J = 22 Hz, *para*-CD), 119.3 (t, J = 23 Hz,*meta*-CD), 63.4 (pent., J = 22.0 Hz, CD_2); MS (ESI) m/z 169 [$(M + Na)^+$, 100], 147 [$(M + H)^+$, 50]; HRMS m/z calcd for $C_7H_2D_7NO_2$ $(M + H)^+$ 147.1142, found 147.1145 (+2.8 ppm error). Spectroscopic data consistent with those reported in the literature for h_7-2,6 pyridine dimethanol.[5]

d_7-2,6-Bis(chloromethyl)pyridine

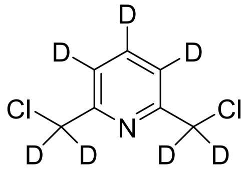

$SOCl_2$ (150 µL, 2.06mmol, 3.0 eq.) was added dropwise to a stirred solution of d_7-pyridine dimethanol (100 mg, 0.69mmol, 1.0 eq.) in THF (10 mL) at rt under N_2. The resulting solution was stirred at 65 °C for 1h and then cooled to rt. The solution was poured into sat. $NaHCO_{3(aq)}$ solution (15 mL) and the two layers were separated. Then, the aqueous layer was extracted with EtOAc (3 x 15 mL) and the combined organic layers were dried (Na_2SO_4) and concentrated under reduced pressure to give the crude product. Purification by flash column chromatography on silica using 95:5 – 8:2 hexane-EtOAc as eluent gaved$_7$-2,6 bis(chloromethyl)pyridine(118 mg, 93%) ascolourless needles, mp73-75 °C (lit.,[6]74 °C); R_f (9:1hexane-EtOAc) 0.2;^{13}C NMR (100.6 MHz, $CDCl_3$) δ156.2, 137.7(t, J = 25.0 Hz, *para*-CD), 121.8 (t, J = 25.0 Hz,*meta*-CD), 45.9 (pent., J = 23.0 Hz, CD_2); MS (ESI) m/z 183 [$(M + H)^+$, 100], 185 [$(M + H)^+$, 65], 187 [$(M + H)^+$, 10]; HRMS m/z calcd for $C_7D_7N^{35}Cl_2$ $(M + H)^+$183.0473, found 147.183.0468 (−0.5 ppm error). Spectroscopic data consistent with those reported in the literature for h_7-2,6-bis(chloromethyl)pyridine.[7]

d_7-2,6-Bis(di-*tert*-butylphosphinomethyl)pyridine di-borane complex[8]

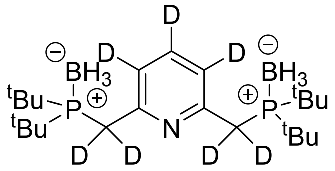

d_7-2,6 bis(chloromethyl)pyridine(70 mg, 0.38 mmol, 1.0 eq.) was added to a stirred biphasic solution of di(*tert*-butyl)phosphine borane complex (182 mg, 1.14 mmol, 3.0 eq.), tetrabutylammnonium bromide (12 mg, 0.038 mmol, 10 mol%), NaOD (8 ml of a 40 wt% solution in D_2O) and toluene (4 mL). The resulting biphasic solution was stirred at rt for 1 h. Then, EtOAc (15 mL) and water (10 mL) were added and the two layers separated. The aqueous layer was extracted with EtOAc (3 x 15 mL) and the combined organic layers were dried ($MgSO_4$) and concentrated under reduced pressure to give the crude product. Purification by flash column chromatography gave on silica using 9:1 – 8:2 hexane-EtOAc d_7-2,6-bis(di-*tert*-butylphosphinomethyl)pyridine di-borane complex(161 mg, 99%) as a white crystalline solid, mp154-156 °C;R_f (8:2 hexane-EtOAc) 0.2;^{1}H NMR (400 MHz, $CDCl_3$)δ7.54 (s, 0.03H, *p*-CH), 7.45 (s, 0.07H, *m*-CH), 3.27 (d, J = 12.0 Hz, 0.06H, CH_2), 1.27 (d, J = 12.5 Hz, 36H, CH_3), 0.92-0.04 (br m, 6H, BH_3); ^{31}P NMR (162 MHz, $CDCl_3$)δ46.5;^{13}C NMR (100.6 MHz, $CDCl_3$) δ 154.3 (Ar), 135.3 (t, J = 25.0 Hz, *para*-CD), 123.3 (t, J = 25.0 Hz, *meta*-CD), 32.7 (d, J = 25.0 Hz, *C*Me$_3$), 28.1 (C*Me*$_3$);MS (ESI) m/z 431 [(M + H)$^+$, 100]; HRMS m/z calcd for $C_{23}H_{42}D_7NP_2B_2$ (M + H)$^+$431.4035, found 431.4046 (+0.9 ppm error).

Note: CD_2 not evident in ^{13}C NMR spectrum. For h_7-2,6-bis(di-*tert*-butylphosphinomethyl)pyridine di-borane complex(see below) CH_2 appears at δ_C 28.7 (d, J = 24.0 Hz, CH_2).

[($C_5D_3N(CD_2P(t\text{-}Bu)_2)_2$)IrH($C_8H_{11}$)($NCCH_3$)]$BF_4$ d_7-1

HBF_4.OEt_2 (1.0 mL) was added to a stirred solution of d_7-2,6-bis(di-*tert*-butylphosphinomethyl)pyridine di-borane complex(30 mg, 0.07 mmol, 1.0eq.) in MeOD (10 mL) at rt under N_2. The resulting solution was stirred at 80 °C for 16 h. Then, the solution was cooled to rt and evaporated under reduced pressure. MeOD (5 mL) and diisopropylaminomethyl-polystyrene (233 mg of 200-400 mesh particle size, extent of labelling: 3 mmol/g, 0.70 mmol, 10.0 eq.) were sequentially added to the residue. The resulting suspension was stirred for 1h atrt. Then, the suspension was filtered by cannula and washed with MeCN (2 x 5 mL). The filtrate was evaporated under reduced pressure. The crude phosphine was dissolved in MeCN (5 mL) and added to [Ir(COD)$_2$]BF_4 (35 mg, 0.07 mmol, 1.0 eq.). The resulting solution was stirred at rt in a glove box for 18 h. Then, the solvent was evaporated under reduced pressure and the residue was washed with MeCN (1 x 5 mL) and hexane (3 x 5 mL) to give **d_7-1**(30 mg, 58 %) as an orange powder. **d_7-4** ^{1}H NMR (400 MHz, CD_3OD) δ -22.3 (t, d, J_{HH} = 14.4 Hz, 13.3 Hz, 1H, Ir-*H*), -18.92 (t, d, J_{HH} = 14.4 Hz, 13.3 Hz 1H, Ir-*H*), 1.04 (m, 18H, PC-(C*H*$_3$)$_3$), 1.24 (m, 18H, PC-(C*H*$_3$)$_3$), 7.64 (d, J_{HH} = 7.8 Hz, 2H, $H_{3,5}$), 7.91 (t, J_{HH} = 7.8 Hz, 1H); ^{31}P NMR (CD_3OD) δ 49.93(s).

d_4-2,6-Bis(di-*tert*-butylphosphinomethyl)pyridine di-borane complex[8]

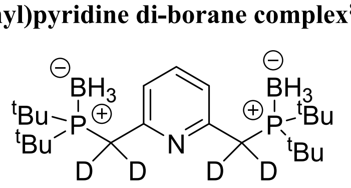

2,6-bis(chloromethyl)pyridine(105 mg, 0.60 mmol, 1.0 eq.) was added to a stirred biphasic solution of di(*tert*-butyl)phosphine borane complex (288 mg, 1.80 mmol, 3.0 eq.), tetrabutylammnonium bromide (19 mg, 0.06 mmol, 10 mol%), NaOD (12 ml of a 40 wt% solution in D_2O) and toluene (6 mL). The resulting biphasic solution was stirred at rt for 16 h. Then, EtOAc (15 mL) and water (10 mL) were added and the two layers separated. The aqueous layer was extracted with EtOAc (3 x 15 mL) and the combined organic layers were dried ($MgSO_4$) and concentrated under reduced pressure to give the crude product. Purification by flash column chromatography gave on silica using 9:1 – 8:2 hexane-EtOAc d_4-2,6-bis(di-*tert*-butylphosphinomethyl)pyridine di-borane complex(251 mg, 98%, 95% D) as a white crystalline solid, mp153-155 °C;R_f (8:2 hexane-EtOAc) 0.2;^{1}H NMR (400 MHz, $CDCl_3$) δ 7.54 (t, J = 8.0 Hz,1H, *p*-CH), 7.45 (d,J = 8.0 Hz,2H, *m*-CH), 3.27 (d, J = 12.0 Hz, 0.22H, CH_2), 1.27 (d, J = 12.5 Hz, 36H, CH_3), 0.92-0.04 (br m, 6H, BH_3); ^{31}P NMR (162 MHz, $CDCl_3$) δ 46.5;^{13}C NMR (100.6 MHz, $CDCl_3$) δ 154.4, 136.0 (Ar), 123.7 (Ar), 32.6 (d, J = 25.5 Hz, *C*Me$_3$), 28.2 (C*Me*$_3$); MS (ESI) m/z 428 [(M + H)$^+$, 100]; HRMS m/z calcd for $C_{23}H_{45}D_4NP_2B_2$ (M + H)$^+$428.3856, found 428.3849 (−0.5 ppm error).

Note: CD_2 not evident in ^{13}C NMR spectrum. For h_7-2,6-bis(di-*tert*-butylphosphinomethyl)pyridine di-borane complex (see below) CH_2 appears at δ_C 28.7 (d, J = 24.0 Hz, CH_2).

[($C_5H_3N(CD_2P(t\text{-}Bu)_2)_2$)IrH($C_8H_{11}$)($NCCH_3$)]$BF_4$ d_4-1

$HBF_4.OEt_2$ (1.0 mL) was added to a stirred solution of d_7-2,6-bis(di-*tert*-butylphosphinomethyl)pyridine di-borane complex**d_4-8** (30 mg, 0.07 mmol, 1.0eq.) in MeOD (10 mL) at rt under N_2. The resulting solution was stirred at 80 °C for 16 h. Then, the solution was cooled to rt and evaporated under reduced pressure. MeOD (5 mL) and diisopropylaminomethyl-polystyrene (233 mg of 200-400 mesh particle size, extent of labeling: 3 mmol/g, 0.70 mmol, 10.0 eq.) were sequentially added to the residue. The resulting suspension was stirred for 1 h atrt. Then, the suspension was filtered by cannula and washed with MeCN (2 x 5 mL). The filtrate was evaporated under reduced pressure. The crude phosphine was dissolved in MeCN (5 mL) and added to [Ir(COD)$_2$]BF_4 (35 mg, 0.07 mmol, 1.0 eq.). The resulting solution was stirred at rt in a glove box for 18 h. Then, the solvent was evaporated under reduced pressure and the residue was washed with MeCN (1 x 5 mL) and hexane (3 x 5 mL) to give **d_4-1**(24 mg, 47 %) as an orange powder. **d_4-4** ^{1}H NMR (400 MHz, CD_3OD) δ -22.3 (t, d, J_{HH} = 14.4 Hz, 13.3 Hz, 1H, Ir-*H*), -18.92 (t, d, J_{HH} = 14.4 Hz, 13.3 Hz 1H, Ir-*H*), 1.04 (m, 18H, PC-(C*H*$_3$)$_3$), 1.24 (m, 18H, PC-(C*H*$_3$)$_3$); ^{31}P NMR (CD_3OD) δ 49.93(s).

h_7-2,6-Bis(di-*tert*-butylphosphinomethyl)pyridine di-borane complex[8]

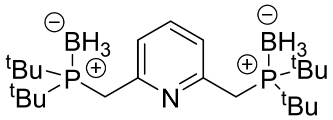

2,6-Bis(chloromethyl)pyridine**7** (53 mg, 0.30 mmol, 1.0 eq.) was added to a stirred biphasic solution of di(*tert*-butyl)phosphine borane complex (144 mg, 0.90 mmol, 3.0 eq.), tetrabutylammnonium bromide (10 mg, 0.03 mmol, 10 mol%), NaOD (6 ml of a 40 wt% solution in H_2O) and toluene (3 mL). The resulting biphasic solution was stirred at rt for 3 h. Then, EtOAc (15 mL) and water (10 mL) were added and the two layers separated. The aqueous layer was extracted with EtOAc (3 x 15 mL) and the combined organic layers were

dried ($MgSO_4$) and concentrated under reduced pressure to give the crude product. Purification by flash column chromatography gave on silica using 9:1 – 8:2 hexane-EtOAc h_7-2,6-bis(di-*tert*-butylphosphinomethyl)pyridine di-borane complex (126 mg, 99%) as a white crystalline solid, mp 153-155 °C;R_f (8:2 hexane-EtOAc) 0.2;^{1}H NMR (400 MHz, $CDCl_3$) δ 7.50 (t, J = 8.0 Hz,1H, *p*-CH), 7.41 (d,J = 8.0 Hz,2H, *m*-CH), 3.25 (d, J = 12.5 Hz, 4H, CH_2), 1.25 (d, J = 12.5 Hz, 36H, CH_3), 0.85-0.02 (br m, 6H, BH_3); ^{31}P NMR (162 MHz, $CDCl_3$) δ 46.9; ^{13}C NMR (100.6 MHz, $CDCl_3$) δ 154.3, 135.7 (Ar), 123.5 (Ar), 32.6 (d, J = 25.5 Hz, *C*Me$_3$), 28.7 (d, J = 24.0 Hz,CH_2), 28.1 (C*Me*$_3$); MS (ESI) m/z 424 [(M + H)$^+$, 100]; HRMS m/z calcd for $C_{23}H_{49}NP_2B_2$ (M + H)$^+$424.3605, found 424.3608 (+3.1 ppm error).

3. Sample preparation

For the polarisation measurements, 2.4 mg of the catalyst was dissolved in 0.6 mL of d_4-methanol and 5 uL of h_5-pyridine added. The solutions were then injected into a 5mm NMR tube fitted with a Young's valve.
A sample was prepared as described above and then pressurised with 3 bar of hydrogen gas before being allowed to stand for 1 hour so activate. $[(C_5H_3N(CH_2P(^tBu)_2)_2)Ir(H)_2(C_6H_5N)]BF_4$ **(4)** was formed in this way. Samples were degassed and pressurised with 3 bar of parahydrogen gas as necessary.

4. Kinetic measurements of the exchange of H_2 and pyridine in 4

The activation parameters for H_2 elimination and pyridine exchange in **4** are present below in table s.1

	$\Delta H^\ddagger$ / kJ mol^{-1}	$\Delta S^\ddagger$ / J K^{-1} mol^{-1}
H_2 exchange	81 ± 4	32 ± 12
Hydride site interchange	88 ± 9	74 ± 32
Pyridine exchange	79 ± 3	50 ± 10

Table S. 1 Hydride interchange, H_2 loss and pyridine activation parameters for **4**.

Temperature / K	Hydrogen dissociation rate constant / s^{-1}	Hydride interchange rate constant / s^{-1}
275	0.066	0.404
278	0.099	0.766
280	0.142	1.161
283	0.201	1.644
285	0.252	2.129
290	0.450	3.87
294	0.705	6.15
298	1.124	9.63

Table S. 2 Hydrogen elimination and hydride site interchange rate constants for 4.

Temperature / K	Rate constants / s^{-1}
260	0.14

264	0.27
268	0.48
272	0.82
276	1.28
280	2.33
284	3.57
288	5.67

Table S 3. Pyridine dissociation rate constants for 4.

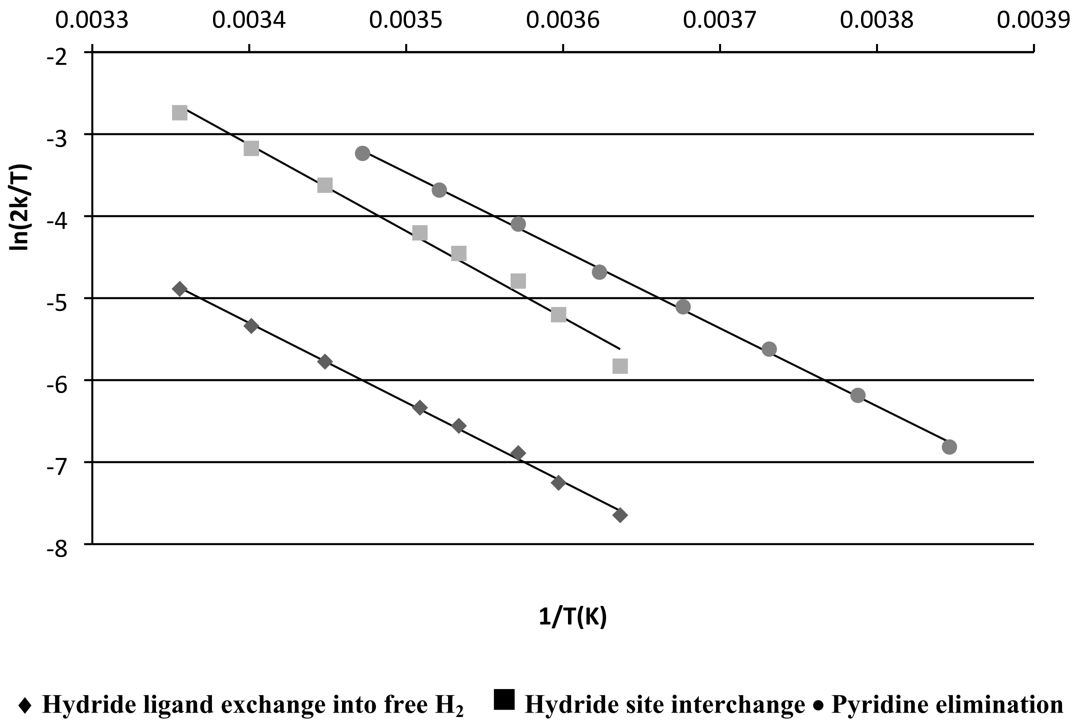

Figure S. 1 Eyring plots for the indicated processes.

5. Effect of hydrogen pressure.

Pressure of Parahydrogen gas	Rate constants / s^{-1}
1 atm	0.43
2 atm	0.70
3 atm	0.90

Table S.4 Hydride ligand exchange into free H_2 rates constants at 290 K as a function of H_2 pressure.

	^{1}H normalised signal integral.		
Time / s	Excited hydride at δ -22.3 / %	Hydrogen gas at δ 4.58 / %	Second hydride at δ -18.9 / %
0.01	92.31	1.74	5.95
0.02	88.53	0.86	10.61
0.04	80.06	1.76	18.18
0.06	72.09	2.51	25.41
0.08	66.59	3.35	30.06
0.1	61.39	4.18	34.43
0.12	58.03	4.94	37.02
0.14	56.59	5.55	37.86
0.16	51.53	7.16	41.31
0.18	51.39	7.46	41.15
0.2	49.38	7.80	42.82
0.25	47.22	9.96	42.81
0.3	44.40	12.11	43.48
0.4	42.26	16.09	41.65
0.5	40.70	17.96	41.34
0.6	38.32	21.78	39.90
0.7	36.84	25.65	37.51
0.8	36.29	30.53	33.18
0.9	34.66	32.89	32.45
1	33.58	34.04	32.38

Table S. 5 Hydride ligand EXSY data at 294 K when the sample is pressurised to a 1 bar hydrogen gas.

	^{1}H normalised signal integral.		
Time / s	Excited hydride at δ -22.3 / %	Hydrogen gas at δ 4.58 / %	Second hydride at δ -18.9 / %
0.01	94.45	0.25	5.29
0.02	88.29	1.28	10.43
0.04	77.71	2.79	19.50
0.06	71.20	4.02	24.78
0.08	63.74	5.37	30.89
0.1	59.78	6.43	33.78
0.12	56.95	7.28	35.76
0.14	52.57	8.23	39.20
0.16	50.60	10.38	39.02
0.18	49.38	10.85	39.76
0.2	48.32	10.40	41.28
0.25	43.89	16.33	39.78
0.3	40.76	19.06	40.18
0.4	37.37	23.92	38.71
0.5	35.58	28.58	35.84
0.6	32.67	34.23	33.10

0.7	30.88	39.40	29.72
0.8	29.11	42.69	28.20
0.9	25.81	47.26	26.93
1	24.17	50.22	25.62

Table S. 6 Hydride ligand EXSY data at 294 K when the sample is pressurised to a 2 bar of hydrogen gas.

	^{1}H normalised signal integral.		
Time / s	Excited hydride at δ -22.3 / %	Hydrogen gas at δ 4.58 / %	Second hydride at δ -18.9 / %
0.01	93.14	2.71	4.15
0.02	85.93	2.46	11.61
0.04	78.62	3.69	17.69
0.06	70.68	5.87	23.45
0.08	63.69	7.56	28.75
0.1	60.41	8.22	31.37
0.12	56.60	8.94	34.47
0.14	51.09	12.02	36.89
0.16	50.60	11.65	37.75
0.18	46.91	13.60	39.49
0.2	45.06	16.31	38.63
0.25	42.54	20.10	37.36
0.3	38.48	22.70	38.82
0.4	35.01	30.29	34.70
0.5	32.51	34.89	32.61
0.6	27.80	43.20	29.00
0.7	28.35	45.39	26.26
0.8	24.59	50.88	24.53
0.9	23.77	52.28	23.96
1	22.53	56.30	21.17

Table S. 7 Hydride ligand EXSY data at 294K when the sample is pressurised to a 3 bar hydrogen gas.

6. Effect of pyridine concentration on the pyridine on hydride ligand exchange rates.

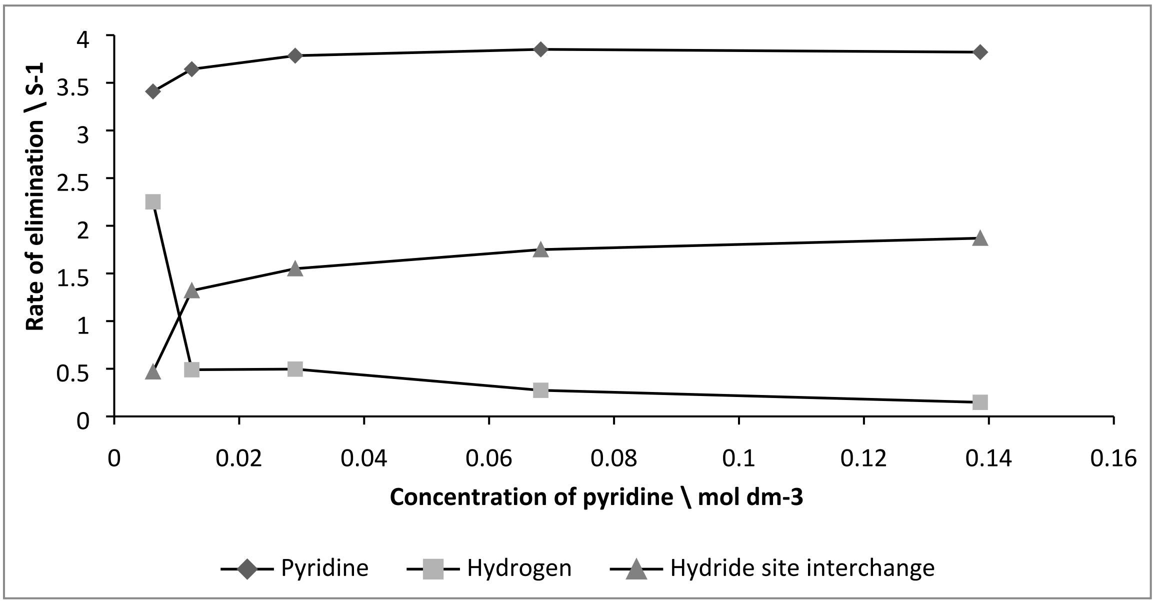

Figure S. 2 Plot of the rate of rates of hydride site exchange into free hydrogen, pyridine and hydride site interchange for 4 as a function of pyridine excess. Data results from EXSY analysis at 290 K and 3 bar H_2 pressure.

7. Quantification of polarisation:

Polarization enhancement factors were calculation using the following equations:

$$Enhancement = \frac{Polarised\ signal}{Thermal\ signal}$$

In a typical experiment, the thermal and polarised spectra where acquired using identical experimental parameters, where the thermal reference spectrum of the sample was only acquired once it has fully relaxed, (typical the sample was left for 10 minutes within the magnet) and before the sample was pressurised with parahydrogen gas. The raw integrals from the thermal and polarised measurement where used to calculate the enchantment level.

8. Effect of transfer field and temperature

In order to monitor the effect of the polarisation transfer field and the samples temperature a sample was prepared as described above and then pressurised with 3 bar of hydrogen gas before being allowed to stand for 1 hour to convert to **4**. The sample was then degassed and pressurised with 3 bar of parahydrogen gas before being immersed in a water bath at the desired temperature. The sample was shaken for approximately10 seconds at either 0.5 or 65 G, and then rapidly inserted into the magnet for measurement. Parahydrogen was replenished after each measurement.

Samples that were polarized in the automated polarizer, used 12 mg of **1** and 0.31 mmol of pyridine in a methanol-d_4 volume of 3 ml. Parahydrogen was introduced into this solution via six inlet tubes at a pressure of 3 bar for 6 s. This solution was then transferred into the Bruker Avance III series 400 MHz spectrometer that was equipped with a TXO flow probe where the flow cell volume was 200 µl by a flow of nitrogen gas. Unless otherwise stated, a single transient was then recorded of the nucleus of interest. Once interrogated, the solution is returned to the polarizing chamber and this process repeated as required. A coil surrounds the reaction chamber such that a magnetic field can be generated in the z direction. This coil was designed to produce static DC fields in the range -150 to 150 x10^{-4} T. Measurements can be made at either +ve or – ve G.

Temperature / K	Enhancement of pyridine		
	Ortho	Para	Meta
275	-6.17	-4.47	5.65
295	-6.36	-5.25	5.62
300	-6.09	-4.62	4.94
305	-5.64	-4.67	4.45
310	-4.66	-4.30	4.09

Table S. 8 Enhancement values for the ortho, meta and para positions of pyridine at 0 G.

Temperature / K	Enhancement of pyridine		
	Ortho	Para	Meta
275	-9.35	-7.50	-0.11
295	-12.26	-10.24	-3.21
300	-13.38	-11.66	-3.04
310	-13.28	-12.20	-4.35

Table S. 9 Enhancement values for the ortho, meta and para positions of pyridine at 65 G.

Catalyst	Field / G	Enhancement of pyridine		
		Ortho	Para	Meta
**d_4-4**	0.5	-6.78	-5.68	5.45
**d_4-4**	65	-16.38	-14.97	-4.60
**d_4-7**	0.5	-9.23	-7.40	7.30
**d_4-7**	65	-16.73	-14.06	-5.23

Table S. 10 Table of enhancement values for ortho, meta and para protons of pyridine when polarised using $[(C_5H_3N(CD_2P(tBu)_2)_2)IrH(C_8H_{11})(NCCH_3)]BF_4$ or $[(C_5D_3N(CD_2P(tBu)_2)_2)IrH(C_8H_{11})(NCCH_3)]BF_4$.

9. Characterisation data for 4

^{1}H -22.3 (t, d, J_{HH} = 14.4 Hz, 13.3 Hz, 1H, Ir-*H*), -18.92 (t, d, J_{HH} = 14.4 Hz, 13.3 Hz 1H, Ir-*H*), 1.04 (s, 18H, PC-(CH_3)$_3$), 1.24 (s, 18H, PC-(CH_3)$_3$), 7.64 (d, J_{HH} = 7.8 Hz, 2H, $H_{3,5}$), 7.91 (t, J_{HH} = 7.8 Hz, 1H) 9.03 (bs, meta). 8.06 (bs, ortho). 7.24 (bs, para). ^{31}P 50.25(s). ^{13}C 36.5 (s, CH_2) 29.74 (s, $PC(CH_3)_3$), 37.84 (Ir-CH_2-P), 122.54 (s, $C_{3,5}$), 139.05 (s, C_4), 165.11 (s, $C_{2,6}$). ESI-MS: m/z 590.28 [M^+] [M^+ -Py]

10. X-Ray crystal structure for 4

Crystals of 4 where grown from a concentrated solution of **4** in methanol. The sample was layered with benzene and the solvent allowed to slowly evaporate. A suitable crystal was selected and mounted on a SuperNova, Single source Eos diffractometer. The crystal was kept at 109.9 K. The structure was solved using Olex2[9], and the olex2.solve[3] structure solution program using charge flipping and refined within the XL[10] refinement package using least squares minimization.

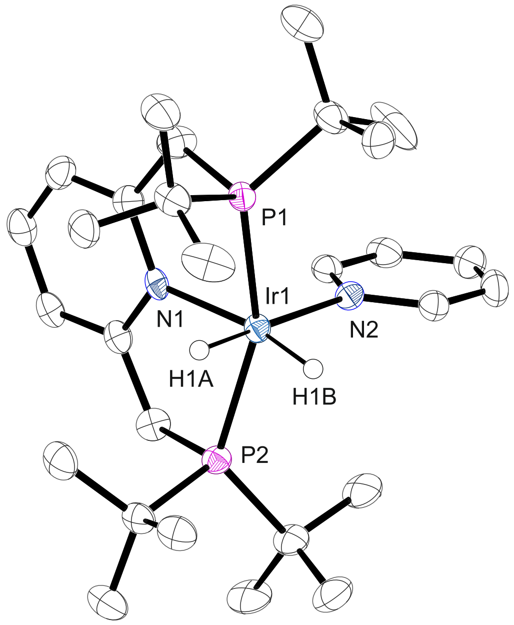

Figure S. 3. ORTEP plot of the cation 4. Ellipsoids are set at 50% probability hydrogen atoms are omitted for clarity.

Crystal Data. $C_{28}H_{50}BF_4IrN_2P_2$, M =755.65, orthorhombic, a = 21.6317(4) Å, b = 12.9870(3) Å, c = 22.4559(5) Å, U = 6308.6(2) Å^3, T = 109.9, space group Pbca (no. 61), Z = 8, μ(Mo Kα) = 4.378, 14358 reflections measured, 5562 unique (R_{int} = 0.0247) which were used in all calculations. The final $wR(F_2)$ was 0.0872 (all data).

Table S. 11 Crystal data and structure refinement for sbd1011

Identification code	sbd1011
Empirical formula	$C_{28}H_{50}BF_4IrN_2P_2$
Formula weight	755.65
Temperature/K	109.9
Crystal system	orthorhombic
Space group	Pbca
a/Å	21.6317(4)
b/Å	12.9870(3)
c/Å	22.4559(5)
α/°	90.00
β/°	90.00
γ/°	90.00
Volume/Å^3	6308.6(2)
Z	8
ρ_{calc}mg/mm^3	1.591
m/mm^{-1}	4.378
F(000)	3040
Crystal size/mm^3	0.1931 × 0.1864 × 0.0554
Theta range for data collection	6.54 to 50.04°
Index ranges	$-23 \le h \le 25$, $-9 \le k \le 15$, $-26 \le l \le 11$
Reflections collected	14358
Independent reflections	5562[R(int) = 0.0247]
Data/restraints/parameters	5562/17/370
Goodness-of-fit on F^2	1.053
Final R indexes [I>2σ (I)]	R_1 = 0.0332, wR_2 = 0.0779
Final R indexes [all data]	R_1 = 0.0473, wR_2 = 0.0872
Largest diff. peak/hole / e Å^{-3}	3.311/-1.158

Table S. 12

Fractional Atomic Coordinates (×10^4) and Equivalent Isotropic Displacement Parameters (Å^2×10^3) for sbd1011. U_{eq} is defined as 1/3 of of the trace of the orthogonalised U_{IJ} tensor.

**Atom**	***x***	***Y***	***z***	**U(eq)**
B1	1911(2)	5600(4)	3953(2)	37.8(18)
F2	2340(5)	6203(8)	4253(5)	52(2)
F2A	2480(6)	6040(20)	4054(12)	52(2)
F3	1913.2(19)	4627(3)	4206.1(18)	51.7(10)
F4	1363.5(17)	6098(3)	4077(2)	57.6(12)
F6	2062(8)	5610(10)	3352(3)	63(4)
F6A	1796(14)	5380(20)	3362(5)	63(4)
C1	-1018(2)	4049(4)	4333(2)	22.3(12)

C2	-972(3)	4966(4)	4650(2)	26.2(12)
C3	-482(3)	5621(5)	4542(3)	29.5(13)
C4	-48(3)	5359(4)	4121(3)	28.6(13)
C5	-108(2)	4443(4)	3807(3)	24.3(12)
C6	-1561(2)	3359(4)	4419(3)	26.4(13)
C7	319(3)	4185(5)	3312(3)	33.4(14)
C8	-1452(2)	4051(4)	2824(2)	24.7(12)
C9	-1847(2)	4489(5)	2414(3)	29.2(13)
C10	-2025(2)	3921(5)	1927(3)	30.6(14)
C11	-1813(3)	2942(5)	1869(3)	32.6(14)
C12	-1432(2)	2534(4)	2302(3)	26.3(12)
C13	-2231(2)	1579(5)	3975(3)	28.5(13)
C14	-2720(3)	1925(6)	4423(3)	40.4(16)
C15	-2239(3)	404(5)	3912(3)	41.0(16)
C16	-2406(3)	2022(6)	3371(3)	42.3(17)
C18	-1137(2)	1316(4)	4835(3)	26.3(13)
C19	-1632(3)	1156(5)	5313(3)	37.3(15)
C20	-863(3)	280(5)	4655(3)	33.3(14)
C21	-626(3)	1974(5)	5116(3)	34.5(14)
C22	528(3)	2877(5)	2286(3)	34.5(14)
C23	-44(3)	3196(9)	1931(3)	71(3)
C24	710(3)	1797(6)	2077(3)	52(2)
C25	1050(3)	3644(5)	2141(3)	41.3(16)
C26	962(3)	2228(5)	3538(3)	30.9(14)
C27	919(3)	2615(6)	4180(3)	40.6(17)
C28	902(3)	1061(5)	3540(3)	44.6(18)
C30	1611(3)	2530(6)	3314(3)	39.7(16)
Ir1	-655.83(9)	2353.18(16)	3458.75(9)	19.27(9)
N1	-592.5(18)	3784(3)	3916.7(19)	19.1(9)
N2	-1240.3(19)	3075(3)	2781(2)	21.3(10)
P1	305.4(6)	2818.8(11)	3101.6(6)	20.8(3)
P2	-1419.3(6)	2027.1(11)	4156.7(6)	21.3(3)

Table S. 13

Anisotropic Displacement Parameters (Å^2×10^3) for sbd1011. The Anisotropic displacement factor exponent takes the form: $-2\pi^2[h^2a^{*2}U_{11}+...+2hka\times b\times U_{12}]$

Atom	U_{11}	U_{22}	U_{33}	U_{23}	U_{13}	U_{12}
B1	44(4)	37(4)	32(4)	-4(3)	13(3)	2(3)
F2	33(4)	63(5)	61(6)	6(4)	15(4)	-15(4)
F2A	33(4)	63(5)	61(6)	6(4)	15(4)	-15(4)
F3	66(3)	37(2)	51(3)	1.4(19)	18(2)	3.5(19)
F4	41(2)	57(3)	74(3)	2(2)	16(2)	9(2)
F6	102(10)	54(7)	33(3)	4(3)	23(4)	32(6)
F6A	102(10)	54(7)	33(3)	4(3)	23(4)	32(6)
C1	22(3)	25(3)	20(3)	1(2)	-3(2)	6(2)
C2	32(3)	28(3)	18(3)	-2(2)	1(2)	6(3)
C3	37(3)	30(3)	22(3)	1(3)	-5(3)	4(3)

C4	30(3)	27(3)	29(3)	3(3)	-9(3)	-4(3)
C5	21(3)	26(3)	26(3)	4(2)	-4(2)	3(2)
C6	24(3)	25(3)	31(3)	1(3)	0(2)	3(2)
C7	31(3)	28(3)	41(4)	-4(3)	11(3)	0(3)
C8	21(3)	32(3)	22(3)	3(2)	1(2)	-3(2)
C9	22(3)	36(3)	30(3)	9(3)	0(3)	0(2)
C10	20(3)	43(4)	29(3)	6(3)	-6(3)	-3(3)
C11	27(3)	49(4)	22(3)	-4(3)	-4(2)	-3(3)
C12	23(3)	32(3)	23(3)	-1(3)	1(2)	-2(2)
C13	17(3)	36(3)	33(3)	4(3)	0(2)	0(2)
C14	17(3)	52(4)	52(4)	8(3)	2(3)	-1(3)
C15	31(3)	41(4)	51(4)	1(3)	-5(3)	-12(3)
C16	23(3)	57(4)	47(4)	13(4)	-10(3)	-11(3)
C18	23(3)	31(3)	25(3)	9(3)	-3(2)	3(2)
C19	33(3)	50(4)	29(4)	10(3)	6(3)	4(3)
C20	33(3)	32(3)	35(4)	9(3)	0(3)	6(3)
C21	31(3)	45(4)	28(3)	7(3)	-6(3)	5(3)
C22	25(3)	54(4)	24(3)	3(3)	4(3)	-1(3)
C23	40(4)	145(9)	26(4)	24(5)	5(3)	9(5)
C24	57(5)	62(5)	35(4)	-19(4)	12(3)	-27(4)
C25	42(4)	49(4)	33(4)	3(3)	14(3)	5(3)
C26	22(3)	42(4)	29(3)	9(3)	-1(2)	6(3)
C27	27(3)	68(5)	26(3)	1(3)	-5(3)	-6(3)
C28	37(4)	41(4)	56(5)	15(3)	15(3)	19(3)
C30	22(3)	64(5)	33(4)	5(3)	3(3)	3(3)
Ir1	15.37(12)	24.19(13)	18.26(13)	-0.45(9)	-1.32(8)	1.99(8)
N1	17(2)	26(2)	14(2)	0.4(19)	-3.9(18)	6.6(19)
N2	16(2)	25(2)	23(2)	0(2)	-0.9(19)	-0.4(18)
P1	15.4(6)	28.0(7)	19.1(7)	-2.3(6)	1.1(5)	1.2(6)
P2	17.1(7)	23.5(7)	23.2(7)	0.3(6)	0.3(6)	0.2(6)

Table S. 14 Selected bond lengths.

Atom	Atom	Length/Å	Atom	Atom	Length/Å
B1	F2	1.389(6)	C12	N2	1.350(7)
B1	F2A	1.373(8)	C13	C14	1.526(8)
B1	F3	1.385(5)	C13	C15	1.532(8)
B1	F4	1.377(5)	C13	C16	1.521(8)
B1	F6	1.389(6)	C13	P2	1.895(6)
B1	F6A	1.383(8)	C18	C19	1.531(8)
C1	C2	1.392(8)	C18	C20	1.524(8)
C1	C6	1.490(8)	C18	C21	1.533(8)
C1	N1	1.356(7)	C18	P2	1.883(6)
C2	C3	1.379(8)	C22	C23	1.528(9)
C3	C4	1.376(8)	C22	C24	1.532(10)
C4	C5	1.389(8)	C22	C25	1.542(9)
C5	C7	1.484(8)	C22	P1	1.895(6)
C5	N1	1.375(7)	C26	C27	1.531(9)

C6	P2	1.853(6)	C26	C28	1.520(9)
C7	P1	1.836(6)	C26	C30	1.541(8)
C8	C9	1.379(8)	C26	P1	1.888(6)
C8	N2	1.350(7)	Ir1	N1	2.128(4)
C9	C10	1.375(8)	Ir1	N2	2.190(4)
C10	C11	1.358(9)	Ir1	P1	2.3092(14)
C11	C12	1.380(8)	Ir1	P2	2.3159(14)

Table S. 15 Selected bond angles

Atom	Atom	Atom	Angle/°	Atom	Atom	Atom	Angle/°
F2A	B1	F2	24.4(8)	C20	C18	C21	108.8(5)
F2A	B1	F3	107.7(13)	C20	C18	P2	110.2(4)
F2A	B1	F4	123.0(12)	C21	C18	P2	107.1(4)
F2A	B1	F6	86.8(9)	C23	C22	C24	107.3(6)
F2A	B1	F6A	113.9(13)	C23	C22	C25	107.9(6)
F3	B1	F2	108.2(6)	C23	C22	P1	108.0(4)
F3	B1	F6	113.9(7)	C24	C22	C25	109.7(5)
F4	B1	F2	102.2(7)	C24	C22	P1	108.9(5)
F4	B1	F3	110.4(4)	C25	C22	P1	114.6(4)
F4	B1	F6	113.2(7)	C27	C26	C30	106.2(5)
F4	B1	F6A	98.0(14)	C27	C26	P1	108.0(4)
F6	B1	F2	108.0(6)	C28	C26	C27	108.6(5)
F6A	B1	F2	134.8(12)	C28	C26	C30	109.4(5)
F6A	B1	F3	101.6(14)	C28	C26	P1	110.1(4)
F6A	B1	F6	27.1(11)	C30	C26	P1	114.4(4)
C2	C1	C6	120.3(5)	N1	Ir1	N2	89.96(16)
N1	C1	C2	121.4(5)	N1	Ir1	P1	83.18(12)
N1	C1	C6	118.1(5)	N1	Ir1	P2	83.02(12)
C3	C2	C1	119.5(5)	N2	Ir1	P1	99.56(11)
C4	C3	C2	119.5(6)	N2	Ir1	P2	97.89(12)
C3	C4	C5	119.8(6)	P1	Ir1	P2	157.68(5)
C4	C5	C7	121.1(5)	C1	N1	C5	118.9(5)
N1	C5	C4	120.8(5)	C1	N1	Ir1	120.7(4)
N1	C5	C7	117.9(5)	C5	N1	Ir1	120.4(3)
C1	C6	P2	112.9(4)	C8	N2	Ir1	123.1(4)
C5	C7	P1	113.7(4)	C12	N2	C8	116.2(5)
N2	C8	C9	123.3(5)	C12	N2	Ir1	120.5(4)
C10	C9	C8	118.9(6)	C7	P1	C22	101.9(3)
C11	C10	C9	119.0(6)	C7	P1	C26	104.3(3)
C10	C11	C12	119.6(6)	C7	P1	Ir1	100.25(19)
N2	C12	C11	123.0(5)	C22	P1	Ir1	125.08(19)
C14	C13	C15	110.3(5)	C26	P1	C22	109.1(3)
C14	C13	P2	114.2(4)	C26	P1	Ir1	113.0(2)
C15	C13	P2	109.6(4)	C6	P2	C13	101.6(3)
C16	C13	C14	107.7(5)	C6	P2	C18	104.8(3)
C16	C13	C15	107.0(5)	C6	P2	Ir1	99.34(18)
C16	C13	P2	107.8(4)	C13	P2	Ir1	124.84(19)

C19	C18	C21	106.9(5)	C18	P2	C13	108.9(3)
C19	C18	P2	114.0(4)	C18	P2	Ir1	113.91(18)
C20	C18	C19	109.7(5)				

Table S. 16 Selected Torsion Angles

A	B	C	D	Angle/°
C1	C2	C3	C4	0.2(8)
C1	C6	P2	C13	-153.8(4)
C1	C6	P2	C18	92.8(4)
C1	C6	P2	Ir1	-25.1(4)
C2	C1	C6	P2	-161.3(4)
C2	C1	N1	C5	-0.3(7)
C2	C1	N1	Ir1	177.8(4)
C2	C3	C4	C5	0.3(8)
C3	C4	C5	C7	174.3(5)
C3	C4	C5	N1	-0.9(8)
C4	C5	C7	P1	163.6(4)
C4	C5	N1	C1	0.9(7)
C4	C5	N1	Ir1	-177.3(4)
C5	C7	P1	C22	151.9(4)
C5	C7	P1	C26	-94.7(5)
C5	C7	P1	Ir1	22.4(5)
C6	C1	C2	C3	-176.6(5)
C6	C1	N1	C5	176.1(5)
C6	C1	N1	Ir1	-5.7(6)
C7	C5	N1	C1	-174.5(5)
C7	C5	N1	Ir1	7.4(6)
C8	C9	C10	C11	0.9(8)
C9	C8	N2	C12	0.6(8)
C9	C8	N2	Ir1	-176.4(4)
C9	C10	C11	C12	0.9(9)
C10	C11	C12	N2	-2.1(9)
C11	C12	N2	C8	1.3(8)
C11	C12	N2	Ir1	178.4(4)
C14	C13	P2	C6	-40.4(5)
C14	C13	P2	C18	69.9(5)
C14	C13	P2	Ir1	-150.5(4)
C15	C13	P2	C6	-164.6(4)
C15	C13	P2	C18	-54.3(5)
C15	C13	P2	Ir1	85.2(5)
C16	C13	P2	C6	79.3(5)
C16	C13	P2	C18	-170.4(4)
C16	C13	P2	Ir1	-30.9(5)
C19	C18	P2	C6	69.1(5)
C19	C18	P2	C13	-39.1(5)
C19	C18	P2	Ir1	176.6(4)

C20	C18	P2	C6	-167.1(4)
C20	C18	P2	C13	84.8(4)
C20	C18	P2	Ir1	-59.6(4)
C21	C18	P2	C6	-49.0(4)
C21	C18	P2	C13	-157.1(4)
C21	C18	P2	Ir1	58.5(4)
C23	C22	P1	C7	-78.9(6)
C23	C22	P1	C26	171.2(6)
C23	C22	P1	Ir1	32.8(7)
C24	C22	P1	C7	164.8(4)
C24	C22	P1	C26	54.9(5)
C24	C22	P1	Ir1	-83.5(4)
C25	C22	P1	C7	41.4(5)
C25	C22	P1	C26	-68.5(5)
C25	C22	P1	Ir1	153.1(4)
C27	C26	P1	C7	46.4(5)
C27	C26	P1	C22	154.6(4)
C27	C26	P1	Ir1	-61.6(5)
C28	C26	P1	C7	164.8(4)
C28	C26	P1	C22	-87.0(5)
C28	C26	P1	Ir1	56.8(5)
C30	C26	P1	C7	-71.6(5)
C30	C26	P1	C22	36.7(6)
C30	C26	P1	Ir1	-179.5(4)
N1	C1	C2	C3	-0.2(8)
N1	C1	C6	P2	22.2(6)
N1	C5	C7	P1	-21.1(7)
N1	Ir1	N2	C8	-6.5(4)
N1	Ir1	N2	C12	176.6(4)
N1	Ir1	P1	C7	-14.0(2)
N1	Ir1	P1	C22	-126.5(3)
N1	Ir1	P1	C26	96.4(2)
N1	Ir1	P2	C6	16.7(2)
N1	Ir1	P2	C13	128.0(3)
N1	Ir1	P2	C18	-94.2(2)
N2	C8	C9	C10	-1.7(8)
N2	Ir1	N1	C1	88.6(4)
N2	Ir1	N1	C5	-93.3(4)
N2	Ir1	P1	C7	74.8(3)
N2	Ir1	P1	C22	-37.7(3)
N2	Ir1	P1	C26	-174.7(2)
N2	Ir1	P2	C6	-72.3(2)
N2	Ir1	P2	C13	39.0(3)
N2	Ir1	P2	C18	176.8(2)
P1	Ir1	N1	C1	-171.8(4)
P1	Ir1	N1	C5	6.4(4)
P1	Ir1	N2	C8	-89.6(4)

P1	Ir1	N2	C12	93.6(4)
P1	Ir1	P2	C6	68.9(2)
P1	Ir1	P2	C13	-179.8(2)
P1	Ir1	P2	C18	-42.0(3)
P2	Ir1	N1	C1	-9.3(4)
P2	Ir1	N1	C5	168.8(4)
P2	Ir1	N2	C8	76.4(4)
P2	Ir1	N2	C12	-100.4(4)
P2	Ir1	P1	C7	-66.2(3)
P2	Ir1	P1	C22	-178.7(3)
P2	Ir1	P1	C26	44.3(3)

Table S. 17: Hydrogen Atom Coordinates (Å×10^4) and Isotropic Displacement Parameters (Å^2×10^3)

**Atom**	***x***	***y***	***z***	**U(eq)**
H2	-1275	5140	4939	31
H3	-445	6248	4757	35
H4	291	5803	4045	34
H6A	-1919	3648	4200	32
H6B	-1669	3341	4847	32
H7A	745	4373	3430	40
H7B	208	4604	2960	40
H8	-1322	4455	3154	30
H9	-1993	5172	2468	35
H10	-2292	4209	1635	37
H11	-1926	2541	1532	39
H12	-1300	1840	2261	32
H14A	-2719	2678	4449	61
H14B	-2626	1631	4814	61
H14C	-3128	1687	4292	61
H15A	-2631	187	3733	62
H15B	-2195	88	4306	62
H15C	-1895	187	3657	62
H16A	-2130	1741	3065	63
H16B	-2366	2773	3381	63
H16C	-2834	1837	3277	63
H19A	-1842	1810	5392	56
H19B	-1437	906	5679	56
H19C	-1934	648	5173	56
H20A	-1189	-155	4485	50
H20B	-688	-59	5006	50
H20C	-538	387	4357	50
H21A	-310	2128	4816	52
H21B	-437	1596	5447	52
H21C	-804	2618	5264	52
H23A	60	3229	1507	106
H23B	-184	3875	2067	106
H23C	-374	2690	1993	106
H24A	383	1308	2183	77

H24B	1097	1592	2271	77
H24C	768	1799	1644	77
H25A	1429	3437	2349	62
H25B	929	4336	2271	62
H25C	1125	3648	1710	62
H27A	1022	3350	4192	61
H27B	1209	2231	4430	61
H27C	497	2514	4328	61
H28A	515	865	3736	67
H28B	1252	760	3757	67
H28C	902	806	3129	67
H30A	1679	2237	2918	60
H30B	1923	2264	3590	60
H30C	1643	3282	3294	60
H1A	-290(20)	1880(50)	3890(20)	46(19)
H1B	-700(40)	1330(30)	3260(40)	90(30)

References

(1) Hermann, D.; Gandelman, M.; Rozenberg, H.; Shimon, L. J. W.; Milstein, D. *Organometallics* **2002**, *21*, 812.
(2) Hull, J. W.; Wang, C. W. *Heterocycles* **2004**, *63*, 411.
(3) Herdering, W.; Krüger, H.-J. *Journal of Labelled Compounds and Radiopharmaceuticals* **1998**, *41*, 301.
(4) Boekelheide, V.; Linn, W. J. *Journal of the American Chemical Society* **1954**, *76*, 1286.
(5) Ueno, Y.; Watanabe, Y.; Shibata, A.; Yoshikawa, K.; Takano, T.; Kohara, M.; Kitade, Y. *Bioorganic & Medicinal Chemistry* **2009**, *17*, 1974.
(6) Paolucci, G.; Fischer, R. D.; Benetollo, F.; Seraglia, R.; Bombieri, G. *Journal of Organometallic Chemistry* **1991**, *412*, 327.
(7) Sprakel, V. S. I.; Elemans, J. A. A. W.; Feiters, M. C.; Lucchese, B.; Karlin, K. D.; Nolte, R. J. M. *European Journal of Organic Chemistry* **2006**, *2006*, 2281.
(8) Lebel, H.; Morin, S.; Paquet, V. *Organic Letters* **2003**, *5*, 2347.
(9) Dolomanov, O. V.; Bourhis, L. J.; Gildea, R. J.; Howard, J. A. K.; Puschmann, H. *J. Appl. Crystallogr* **2009**, *42*, 339.
(10) Sheldrick, G. M. *Acta. Crystallogr. Sect. A* **2008**, *64*, 112.
